# Pulmonary Hypertension and Right Heart Dysfunction in Chronic Lung Disease

**DOI:** 10.1155/2014/739674

**Published:** 2014-07-24

**Authors:** Amirmasoud Zangiabadi, Carmine G. De Pasquale, Dimitar Sajkov

**Affiliations:** ^1^Australian Respiratory and Sleep Medicine Institute, Flinders Medical Centre, Bedford Park, Adelaide, SA 5042, Australia; ^2^Department of Cardiology, Flinders Medical Centre, Bedford Park, Adelaide, SA 5042, Australia

## Abstract

Group 3 pulmonary hypertension (PH) is a common complication of chronic lung disease (CLD), including chronic obstructive pulmonary disease (COPD), interstitial lung disease, and sleep-disordered breathing. Development of PH is associated with poor prognosis and may progress to right heart failure, however, in the majority of the patients with CLD, PH is mild to moderate and only a small number of patients develop severe PH. The pathophysiology of PH in CLD is multifactorial and includes hypoxic pulmonary vasoconstriction, pulmonary vascular remodeling, small vessel destruction, and fibrosis. The effects of PH on the right ventricle (RV) range between early RV remodeling, hypertrophy, dilatation, and eventual failure with associated increased mortality. The golden standard for diagnosis of PH is right heart catheterization, however, evidence of PH can be appreciated on clinical examination, serology, radiological imaging, and Doppler echocardiography. Treatment of PH in CLD focuses on management of the underlying lung disorder and hypoxia. There is, however, limited evidence to suggest that PH-specific vasodilators such as phosphodiesterase-type 5 inhibitors, endothelin receptor antagonists, and prostanoids may have a role in the treatment of patients with CLD and moderate-to-severe PH.

## 1. Introduction

Pulmonary hypertension (PH), defined as an elevated mean pulmonary arterial pressure (mPAP) ≥25 mmHg, is a common complication of chronic lung disease (CLD). PH often progresses to right heart failure (RHF), with initial compensatory right ventricular (RV) hypertrophy becoming overwhelmed by increased systolic requirements, whilst left ventricular (LV) systolic function remains preserved. The term “cor pulmonale” has been used to describe this form of RHF and hypertrophy. It is a progressive condition, associated with increased mortality in CLD.

The World Health Organization (WHO) has classified PH into five groups based on their pathological and haemodynamic characteristics [1]. This review will focus on group 3 PH secondary to lung diseases and/or hypoxia and its effects on RV. Patients with chronic obstructive pulmonary disease (COPD), interstitial lung disease (ILD), and sleep-disordered breathing (SDB) or obstructive sleep apnoea (OSA) account for majority of the cases in this group [2].

Updated Classification of Pulmonary Hypertension (5th WSPH Nice 2013 [1]) is as follows.

1. Pulmonary arterial hypertension.
  - Idiopathic PAH.
  - Heritable PAH.
    - BMPR2.
    - ALK-1, ENG, SMAD9, CAV1, and KCNK3.
    - Unknown.
  - Drug and toxin induced.
  - Associated with:
    - connective tissue disease;
    - HIV infection;
    - portal hypertension;
    - congenital heart diseases;
    - schistosomiasis.
2. (1*α*) Pulmonary venoocclusive disease and/or pulmonary capillary haemangiomatosis.
3. (1*β*) Persistent pulmonary hypertension of the newborn (PPHN).
4. Pulmonary hypertension due to left heart disease.
  - Left ventricular systolic dysfunction.
  - Left ventricular diastolic dysfunction.
  - Valvular disease.
  - Congenital/acquired left heart inflow/outflow tract obstruction and congenital cardiomyopathies.
5. Pulmonary hypertension due to lung diseases and/or hypoxia.
  - Chronic obstructive pulmonary disease.
  - Interstitial lung disease.
  - Other pulmonary diseases with mixed restrictive and obstructive pattern.
  - Sleep-disordered breathing.
  - Alveolar hypoventilation disorders.
  - Chronic exposure to high altitude.
  - Developmental lung diseases.
6. Chronic thromboembolic pulmonary hypertension (CTEPH).
7. Pulmonary hypertension with unclear multifactorial mechanisms.
  - Hematologic disorders: chronic haemolytic anaemia, myeloproliferative disorders, and splenectomy.
  - Systemic disorders: sarcoidosis, pulmonary histiocytosis, and lymphangioleiomyomatosis.
  - Metabolic disorders: glycogen storage disease, Gaucher's disease, and thyroid disorders.
  - Others: tumoral obstruction, fibrosing mediastinitis, and chronic renal failure.
  - Segmental PH.

BMPR: bone morphogenic protein receptor type II; CAV1: caveolin-1; ENG: endoglin; HIV: human immunodeficiency virus; PAH: pulmonary arterial hypertension.

## 2. Prevalence

The prevalence of PH in patients with CLD has been debated. One of the reasons for skewed reporting is the selection bias. Most research into PH has been conducted in selected patient populations with advanced lung disease and the reported prevalence, therefore, may not be applicable to patients with less severe disease.

RV dysfunction is common in patients with COPD and more pronounced in the presence of PH [3]. In a cohort study of patients with COPD, Freixa et al. [3] found echocardiographic enlargement of the RV and PH in 30% and 19% of patients, respectively. PH was more common in those with severe COPD (33%).

Impaired left ventricular (LV) diastolic filling is another common complication of COPD with the prevalence as high as 90% in severe stable disease [4]. LV diastolic dysfunction can contribute to PH and is independently associated with reduced exercise tolerance.

PH in COPD patients is closely associated with patient age and severity of airway obstruction [5]. In a large multicentre study of patients with severe emphysema (forced expiratory volume in one second (FEV_1_) < 45%) who underwent right heart catheterisation, the prevalence of PH was 38% with the majority showing mild to moderate PH with normal cardiac output [6]. In patients with severe COPD PH prevalence of up to 60% has been reported [7]. An mPAP of 20 mmHg has been considered the upper limit of normal in CLD patients, which is lower than the 25 mmHg required for clinical diagnosis of PH [8]. Most stable COPD patients have mild to moderate PH with mPAP between 25 and 30 mmHg [9].

Estimated prevalence of PH in patients with idiopathic pulmonary fibrosis (IPF) awaiting lung transplant varies between 31% and 85% [10, 11]. In one study of 212 patients with ILD screened by echocardiography and/or right heart catheterisation 14% were found to have PH, of which 6% had severe PH defined as mPAP ≥ 35 mmHg [12].

The prevalence of PH in patients with OSA is estimated to range from 17 to 52% [13]. In one study of 27 patients with OSA 41% had mildly elevated PA pressures (mPAP = 26 mmHg), in the absence of cardiac or pulmonary disease [14]. Higher prevalence of up to 70% has been reported in unselected patients [15]. However, considering mild severity of PH in patients with OSA, screening of asymptomatic patients is not recommended.

PH often occurs in otherwise healthy high altitude residents at various locations. High altitude PH (HAPH) is a consequence of excessive hypoxic pulmonary vasoconstriction and pulmonary vascular remodelling, pathophysiologic mechanisms shared with primary pulmonary diseases. Its prevalence varies between 5 and 18% in those living at ≥3000 meters and may be more common in children than adults [16–18].

## 3. Effect of CLD on Heart Function

Autopsy evidence of cor pulmonale has been found in more than 40% of patients with CLD [23]. Postmortem RV hypertrophy is observed in two-thirds of patients with chronic bronchitis [24] and one-third of patients with emphysema [25]. Hilde et al. [26] showed that cardiac complications of CLD, including reduced RV systolic function and RV hypertrophy, start early in the course of the disease even at subclinical levels of raised mPAP. They observed that even a slight increase in mPAP at rest was associated with a substantial adaptive increase in RV hypertrophy and dilatation. These changes are progressive and could lead to RV impairment. In fact an elevated mPAP > 25 mmHg, indicating that at least 50% of the pulmonary vascular bed has been damaged, is a late marker of lung vascular remodelling with significant impact on the RV [26]. RV hypertrophy in stable COPD is associated with preserved systolic function [27]; however, COPD exacerbation and progression are frequently associated with RV dilatation and failure [28, 29].

Using cardiac magnetic resonance (CMR), Sato et al. [30] assessed RA structure and function in PH. They observed increased size, decreased reservoir function, and increased conduit function associated with PH. RV ejection fraction (RVEF) increases in patients with mild to moderate PH but decreases in advanced PH.

Several mechanisms may contribute to the development of RV failure in CLD. Pulmonary hyperinflation in COPD affects pulmonary haemodynamics and consequently heart size and function. Hyperinflation can reduce the intrathoracic volume and heart filling pressures and mechanically compress the ventricles [31]. Expiratory airflow limitation in COPD patients leads to expiratory blood flow limitation in the pulmonary circulation and subsequently impairment of resting and exercise stroke volume [32]. It has been shown that COPD patients have smaller pulmonary vein dimensions and reduced LV filling compared to normal individuals [33]. A direct correlation between impaired LV filling and cardiac output and hyperinflation in COPD has also been observed on CT imaging [34]. In the setting of severe PH the interventricular septum flattens or even bows to the left to accommodate pressure overload. This negatively impacts LV function, resulting in lower stroke volume and cardiac output [35]. RV ischaemia in the presence of decreased perfusion pressure and increased oxygen demand will further worsen RV function.

## 4. Pathophysiology of PH in CLD

**Figure 1:**
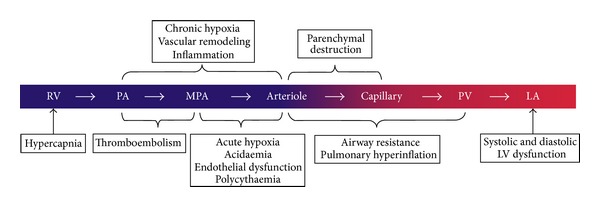
Schematic illustration of the site of action for each of the pathogenic mechanisms for pulmonary hypertension in COPD. LA: left atrium; LV: left ventricle; MPA: muscular pulmonary artery; PA: pulmonary artery; PV: pulmonary vein; RV: right ventricle [19]. Reproduced with permission of the European Respiratory Society [60].

The pathophysiology of PH in CLD is complex and multifactorial. Increased tone of small pulmonary arteries is the result of hypoxic pulmonary vasoconstriction, capillary endothelial and smooth muscle proliferation, and muscularization of previously nonmuscular arteries [19]. Hypoxia and chronic inflammation are the main factors driving vasoconstriction, vascular remodelling, and PH [36, 37] (Figure 1).

**Figure 2:**
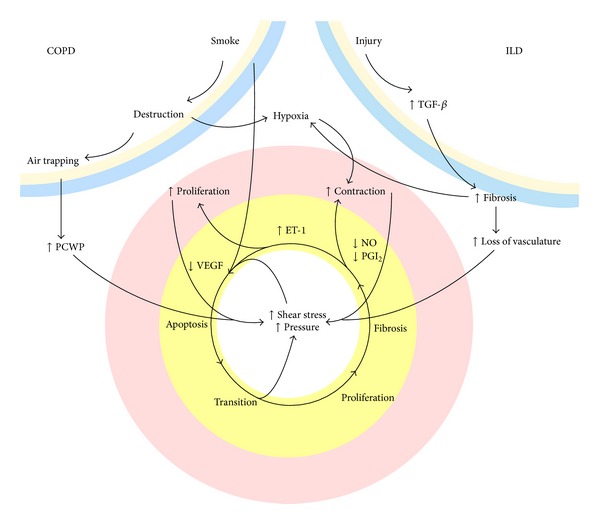
Mechanisms contributing to PH in COPD. BMSC: bronchial smooth muscle cells, VSMC: vascular smooth muscle cells, VEGF: vascular endothelial growth factor, ET-1: endothelin-1, NO: nitric oxide, PGI_2_: prostacyclin, TGF-*β*: tumor growth factor-*β*, PCWP: pulmonary capillary wedge pressure. Yellow: endothelium, pink: vascular smooth muscle cells blue/sand: respiratory wall [20]. Reproduced with permission.

Acute alveolar hypoxemia induces vasoconstriction to divert nonoxygenated blood towards better ventilated areas, preserve the ventilation to perfusion ratio, and maintain oxygen saturation in the blood [38]. Chronic hypoxemia, however, can cause vascular remodelling and increased PVR [39]. The mechanism by which chronic hypoxemia results in PH is not fully understood; however, studies have shown that chronic hypoxia can cause endothelial dysfunction which subsequently prevents homeostatic inhibitory effects of prostacyclin and nitric oxide on vascular remodelling [19]. The mitochondria in vascular smooth muscle cells are the oxygen sensors that initiate this process [36]. Mitochondria-derived reactive oxygen species (ROS) increase intracellular calcium concentration in pulmonary arterial smooth muscle cells and induce vasoconstriction [37]. Chronic hypoxia also causes a systemic inflammatory response [40]. Hypoxia-induced mitogenic factor (HIMF), which is released from lung macrophages during hypoxia, is linked to angiogenesis via VEGF [40]. In addition, overexpression of interleukin-6 (IL-6) during hypoxemia can induce cell proliferation and reduced apoptosis on vascular endothelium, smooth muscles cells, and fibroblasts, promoting vascular remodelling [41] (Figure 2).

A recent study showed that the adenosine A2B receptor (ADORA2B) and hyaluronan contribute to vascular remodelling and the development of PH in COPD [42]. The inhibition of ADORA2B was demonstrated to attenuate PH hallmarks in an animal model of airspace enlargement and vascular remodelling. Increased arginase expression in COPD is another culprit in the development of PH [43]. Arginase inhibitors in guinea pig models of COPD can effectively reverse its role. These findings may provide new targets in the development of treatments for PH in COPD.

The role of hypercarbia and parenchymal destruction in the pathogenesis of PH is less understood. In the presence of severe PH other comorbidities such as sleep-disordered breathing, LV diastolic dysfunction, and thromboembolism need to be excluded [19].

The mechanism of PH development in ILD is still the subject of investigation with many factors, aside from hypoxemia and parenchymal tissue loss, believed to play a role [44, 45]. For example, epithelial damage in IPF has been shown to cause fibroblast activation and the release of mediators (e.g., TGF-*α*) leading to endothelial apoptosis [46]. This in turn results in decreased vascular density as well as release of pulmonary vascular smooth muscle (PVSMC) growth factor which promotes vascular remodelling [47]. In systemic sclerosis, autoantibodies such as antifibrillin and anti-EC antibodies have also been found to promote endothelial apoptosis [48]. Endothelin-1 (ET-1) is a vasoconstrictor and a growth factor for PVSMC. Endothelial dysfunction with reduced levels of nitric oxide and prostacyclins and increased levels of endothelin-1 and thromboxanes may thus contribute to the development of PH [49, 50]. Ventetuolo et al. revealed higher plasma levels of ET-1 in patients with IPF to be associated with higher PAP and possibly higher PVR [51]. Increased levels of ET-1 have also been found in the bronchoalveolar lavage fluid of patients with severe sarcoidosis and PH [52], with no evidence of endothelial injury [46, 53]. In a large cohort of sarcoidosis patients with PH, Rapti et al. found pulmonary fibrosis and LV diastolic dysfunction to be the main pathogenic mechanisms of PH development [54].

In patients with OSA the main mechanisms for development of PH are repetitive nocturnal hypoxemia, increased sympathetic tone, and wide swings in intrathoracic pressure [55, 56]. Cyclical intermittent hypoxemia can cause overexpression of ET-1 in pulmonary arteries [57]. Our data indicates that pulmonary vasoconstriction rather than remodelling is the main cause of elevated PVR in this group of patients, with reversibility seen after 6 months of continuous positive airway pressure (CPAP) treatment [14, 58].

## 5. Prognosis

RV failure is the end point of all forms of PH. The thin walled crescent-shaped RV adapts to PH through hypertrophy to maintain cardiac output in the face of high afterload. Eventually, however, it will become overwhelmed and fail leading to the syndrome of RHF. It is failure of the RV that leads to increased morbidity and mortality in PH of all subtypes [59].

In lung transplant candidates, RV function during exercise, measured by equilibrium radionuclide angiography (ERNA), is a stronger predictor of outcome than RV function at rest [60]. The inability to augment RVEF during exercise is a sign of inadequate RV reserve. In patients with acute decompensated heart failure the combination of PH and RV dysfunction is a poor prognostic factor with adjusted 1-year mortality hazard ratio (HR) of 2.4 compared with patients with no RVF or PH [61].

**Figure 3:**
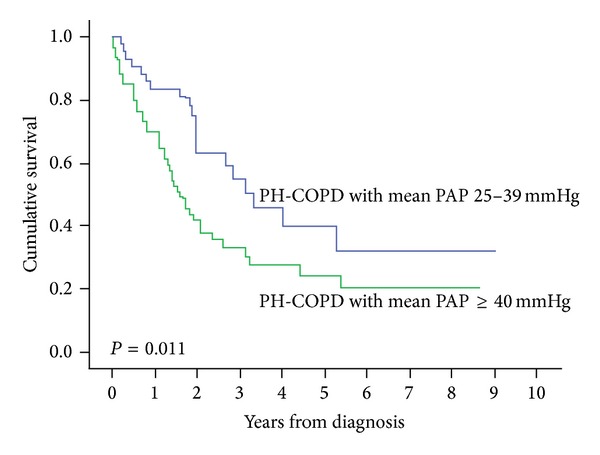
Cumulative survival from date of diagnosis in pulmonary hypertension associated with COPD by mean PAP [21]. Reproduced with permission.

Several studies have shown that COPD patients with PH have a reduced survival [21, 62–64]. In a large cohort study of patients with COPD and severe PH, one-year survival was 70% and 3-year survival 33%, significantly worse than the 83% and 55%, respectively, seen in COPD patients with mild to moderate PH [21] (Figure 3). In this study an mPAP of 40 mmHg was a determinant of survival with age, DLCO, mixed venous oxygen saturation, and World Health Organization (WHO) functional class, all independent predictors. Cuttica et al. [62] also demonstrated that COPD patients with PH were at greater risk of dying on the transplant list.

**Figure 4:**
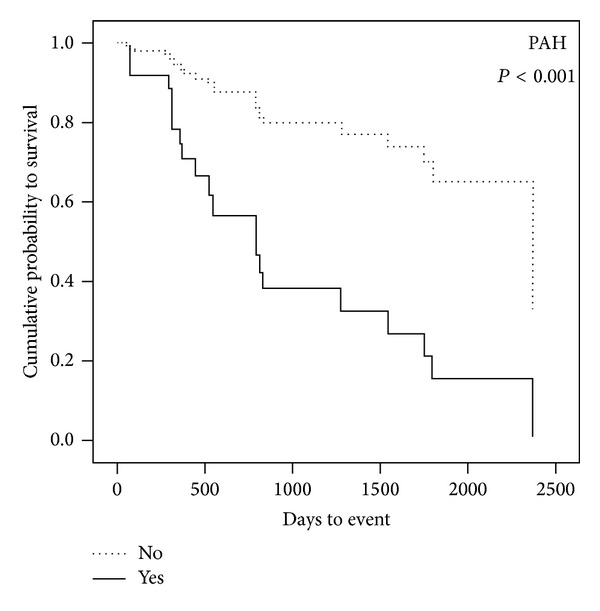
PH as a predictor of survival in patients with IPF [22]. Reproduced with permission.

Similarly PH has been associated with reduced survival in patients with ILD. On long-term follow-up of patients with IPF, Hamada et al. [65] showed a reduced 5-year survival in patients with PH (16.7%) as compared to patients without (62.2%). Lettieri at al. [22] also found that PH was more common in nonsurvivors of IPF, with neither LV dysfunction nor transplant changing outcomes (Figure 4).

Data regarding outcome of PH in patients with OSA is scarce. Minai et al. [15] found in their cohort of patients with OSA that 1-, 4-, and 8-year survival rates in patients with PH were 93%, 75%, and 43%, respectively, as compared to 100%, 90%, and 76% in those without PH.

## 6. Diagnosis

Symptoms of PH and RHF are nonspecific. Dyspnoea on exertion is hard to differentiate from that found in chronic lung disease. Other symptoms such as loud snoring and higher BMI are associated with higher mPAP in COPD patients [6]. Signs of PH on examination include left parasternal heave, palpable second heart sound, systolic murmur of tricuspid regurgitation, and diastolic murmur of pulmonary insufficiency. Severe PH with RHF can present with elevated jugular venous pressure, pulsatile, tender hepatomegaly, and peripheral oedema [66], although the latter is not specific and can be present in patients with CLD without PH [67]. In a study of 95 patients with COPD, PaO_2_ < 71 mmHg was also associated with the presence of PH with a sensitivity of 76% [68].

In addition to hypoxemia, the degree of pulmonary function impairment is important and correlates to the severity of PH. Minai et al. showed that an increase in mPAP in COPD patients was associated with a decrease in FEV_1_% and diffusion capacity for carbon monoxide (DLCO%). The authors also found higher postbronchodilator residual volume to be associated with PH in COPD patients [6]. However, in a subset of patients with diffuse emphysema and severe PH spirometry does not correlate with PH severity. Such patients usually present with normal spirometry and lung volumes but severely reduced DLCO and hypoxemia [69]. A severely reduced DLCO is an independent predictor for PH in sarcoidosis and a sensitive negative prognostic factor for PH in systemic sclerosis and idiopathic pulmonary fibrosis [54].

PH is associated with limited exercise capacity in patients with COPD. It has been shown that these patients have shorter 6MWT distance after adjustment for lung function [70]. 6MWT distance less than 400 meters is an independent predictor of death and clinical deterioration in patients with nonclass 1 PH [71].

Brain natriuretic peptide (BNP) and its N-terminal fragment (NT Pro-BNP) are secreted in response to elevated cardiac wall stress. Plasma levels of BNP and NT Pro-BNP are elevated in patients with CLD and associated with RV failure. Levels are significantly higher in patients with severe COPD (GOLD III and IV) and in the presence of PH, even in asymptomatic patients [72]. The high sensitivity (85%) and specificity (88%) [73] of these serum markers make them useful to rule out significant PH when levels are not elevated [74].

The tumour marker CA-125 may also assist identification of RV failure in COPD patients. RV failure and consequently congestion of splanchnic mesothelium can increase CA-125 levels. In one study by Yilmaz et al. [75] patients with COPD had significantly higher levels of CA-125 compared to controls, with levels correlated to echocardiographic parameters of RV failure.

Radiographic evaluation of suspected PH begins with chest radiography (CXR). Signs of PH on CXR include isolated enlargement of the RV, right descending pulmonary artery diameter >16 mm, and pruning of the pulmonary vessels. Miniati et al. [76] studied the accuracy of these findings in predicting PH and found their weighted sensitivity to be 96.9% and weighted specificity to be 99.8%. Computed tomography (CT) of the chest has also been used to evaluate the severity of PH. In a multicentre observational trial of 3,464 patients with COPD, Wells et al. found a pulmonary artery to aorta diameter (PA : A) ratio ≥ 1 to be associated with severe exacerbations of COPD [77]. This ratio correlated linearly with mPAP but not with systolic pulmonary artery pressure (SPAP) [78].

Doppler echocardiography (DE) is a noninvasive method to measure estimated SPAP and RV function. It can be technically challenging, however, in the setting of hyperinflation and poor acoustic windows in patients with CLD. The usual DE parameters to screen for PH are tricuspid regurgitation pressure gradient (TR), right atrial and ventricular size, tricuspid annular plane systolic excursion (TAPSE), and transverse view eccentricity [79]. There is no consensus regarding the upper normal limit of SPAP; however, values above 35 mmHg are considered to make diagnosis of PH possible with values above 55 mmHg making a diagnosis of PH likely [80]. In a study of 374 patients with severe COPD and ILD, the positive and negative predictive values of an elevated SPAP > 45 mmHg for diagnosing PH were only 52% and 87%, respectively, when compared with the results of right heart catheterization (RHC) [81]. Similar results were achieved in another study by Andersen et al. [74]. Despite having a low predictive value, high mortality and low exercise capacity in patients with positive screening for PH makes DE a valuable test which could lead to further investigations [74]. Newer echocardiographic techniques are more promising with 3D evaluation of RV ejection fraction, tissue Doppler imaging (TDI) velocities, and strain making it possible to identify RV impairment at an earlier stage [26].

The gold standard for measuring PAP is RHC. Based on updated recommendations from the Cologne Consensus conference 2011, at least 2 of the following criteria should be met in order to diagnose PH in patients with CLD [66]:

1. mPAP > 35 mmHg;
2. mPAP ≥ 25 mmHg with limited cardiac output (CI < 2.0 L/min/m^2^);
3. pulmonary vascular resistance (PVR) > 480 dyn*·*s*·*cm.

RHC is an invasive and costly procedure requiring specialized skills and radiation exposure. Given that the incidence of PH in CLD is modest and current therapeutic options for idiopathic PH are not indicated in these patients, RHC is not routinely recommended in this population. RHC is, however, performed in preparation for lung transplantation and in patients with severe RV failure disproportionate to their underlying CLD. The latter comprises an unusual pattern of cardiopulmonary abnormalities that have been described in patients with CLD who present with severe PH and other signs including mild to moderate airway obstruction, severe hypoxemia, hypocapnia, and a very low DLCO. Such presentations are believed to be a “vascular phenotype” characterised by presence of obstructive airways disease and fibrosis [82].

## 7. Treatment

In general, treatment of the underlying CLD is the mainstay of management for RV dysfunction. Other comorbidities such as LV dysfunction and pulmonary embolism should also be addressed and treated as they will impact on RV function and consequently hasten decline to RHF and death. Salt and water restriction are commonly implemented strategies in any form of heart failure. Diuretics are recommended for RHF and associated fluid overload with close monitoring of renal function and serum electrolytes. Their role is one of decongestion and symptom relief.

Unlike its left heart counterpart there is a remarkable paucity of proven therapy for RHF. While there are mechanistic similarities in neurohormonal modulation, such as elevated sympathetic activity, renin-angiotensin-aldosterone system (RAAS) activation, and maladaptive cardiac remodelling in both forms of failure, the cornerstone treatments of LHF (i.e., ACE inhibitors, *β* adrenergic receptor blockers, and aldosterone antagonists) have no proven effects in RHF [59]. In the subgroup of CLD there is again some evidence of RAAS activation [83] consistent with a failing heart; however, there are no studies showing benefit of therapy aimed at this maladaptive compensatory neurohormonal activation.

There is limited evidence to suggest that PH-specific vasodilators such as phosphodiesterase-type 5 (PDE-5) inhibitors, endothelin receptor antagonists (ERA), and prostanoids have a role in the treatment of patients with CLD. On the contrary, they may nonselectively dilate the vessels in hypoventilated areas of the lung and worsen hypoxemia [38, 84]. As such, standard therapy with smoking cessation, long-term oxygen therapy (LTOT), bronchodilators, inhaled steroids, and pulmonary rehabilitation remain the focus of treatment in these patients [85]. PH-specific therapies for COPD patients are only considered empirically when PH is persistent despite optimal COPD management and LTOT, or when PH is believed to be disproportionate to the underlying lung disease. The evidence for their use in CLD is scarce and consists of case reports and small randomised controlled trials (RCT).

In most ILD, the main treatment approach to PH is to treat the underlying parenchymal lung disease. Due to the rarity of other forms of ILD, data regarding the effect of PH-specific therapies in this subgroup has largely come from study populations with idiopathic pulmonary fibrosis. Currently, immunosuppression is the predominant treatment strategy, as the value of using PH-specific therapy in this group of patients has not been established.

### 7.1. Positive Pressure Ventilation for Obesity Hypoventilation Syndrome and Obstructive Sleep Apnoea

Management of patients with PH in the setting of OSA and obesity hypoventilation syndrome (OHS) is again aimed at treating the underlying disease. In a study of 20 patients with OSA, treatment with CPAP over a 4 month period reduced the mean PAP by 13.9 mmHg [86]. Arias et al. [13] also demonstrated significant improvement in pulmonary artery pressures with effective CPAP therapy. The reduction of PAP following CPAP treatment is associated with improved pulmonary endothelial function through elimination of intermittent hypoxemia. While current data suggests improvement in PH with CPAP therapy, the clinical significance of this improvement remains unclear particularly with mild to moderate PH observed in most patients with OSA without lung or heart disease.

### 7.2. Long-Term Oxygen Therapy (LTOT)

The only therapy that has demonstrated a survival advantage in patients with coexistent COPD and PH is LTOT. The Medical Research Council (MRC) study showed that 15 hours of daily oxygen therapy in COPD patients with a resting PaO_2_ < 55 mmHg or < 59 mmHg and signs of RV failure or polycythaemia reduced 5-year mortality from 67% to 45% [87]. It also reduces pulmonary artery pressure, however, not significantly in those with severe PH [88–90]. Downsides to LTOT include its expense and associated adverse events such as CO_2_ retention or burns, particularly where patients continue to smoke [91–93]. The adherence to treatment is also variable ranging between 45 to 70% [94, 95].

### 7.3. Nitric Oxide (NO)

Endogenous NO produced from oxygen and L-arginine facilitates smooth muscle relaxation and decrease pulmonary vascular resistance. As NO avidly binds to haemoglobin; this effect is localized to the lungs with no increase in systemic vasodilatation. Inhaled NO has been used to improve haemodynamics and exercise capacity in secondary PH. Pulsed inhalation of mixed nitric oxide and oxygen, when compared to LTOT in stable COPD patients with PH, improved mPAP and PVR [83]. Its use, however, is expensive, logistically difficult, and associated with methemoglobinemia, greatly reducing its applicability.

### 7.4. PDE-5 Inhibitors

The enzyme PDE-5 catabolises cyclic guanosine monophosphate (cGMP), the secondary messenger of NO which exerts its effects on pulmonary arterial smooth muscle cells (PASMC). By inhibiting this enzyme PDE-5 inhibitors increase c-GMP concentration in PASMC following NO stimulation thus resulting in vasodilatation [2]. Experimental models of fixed PH (pulmonary artery banding) have demonstrated a positive effect on RV remodelling with PDE-5 inhibitor therapy implying a possible direct effect on the RV [96]. Sildenafil, a PDE-5 inhibitor, may be preferred to other vasodilator agents, particularly in patients with severe COPD, PH, and poor RV function, as its hemodynamic effects are likely to be selective to pulmonary circulation. PDE-5 inhibition with sildenafil attenuates the rise in PAP and vascular remodelling when given before chronic exposure to hypoxia and when administered as a treatment during ongoing hypoxia-induced PH [97]. A randomized trial in 20 patients with COPD-associated PH demonstrated that sildenafil improved pulmonary haemodynamics both at rest and during exercise. This effect was also associated, however, with mild to moderate worsening of gas exchange at rest due to increased V/Q mismatch [98]. In another study neither stroke volume nor exercise capacity were improved by 3 months of sildenafil therapy [99].

A few studies have evaluated the role PDE-5 inhibitors in patients with ILD. In a small open-label prospective trial, Collard et al. [100] showed a 57% improvement in 6MWT following treatment with sildenafil in patients with IPF. In a randomized trial examining the effect of sildenafil on 6MWD in patients with advanced IPF and a DLCO below 35%, treatment was associated with preservation of exercise capacity in patients with RV systolic dysfunction as compared with placebo. Sildenafil has also been found to improve quality of life in these patients [101]. In a further study, sildenafil and tadalafil were used for the treatment of PH in IPF and hypersensitivity pneumonitis (HP) [102]. After a minimum 3 months of treatment, an increase in cardiac index (CI) and decrease in PVR were observed but 6MWT and BNP level did not change significantly.

### 7.5. Endothelin (ET) Receptor Antagonists

ET1 secreted from endothelial cells is a powerful vasoconstrictor inducing calcium release from the sarcoplasmic reticulum and causing smooth muscle contraction. It applies its effect on PASMCs via two receptors, endothelin receptor A and B (ETa, ETb). Overexpression of these receptors can be found in the pulmonary arteries of smokers as compared to nonsmoking patients [103].

Bosentan, a nonselective endothelin receptor antagonist, has been shown to attenuate the overexpression of endothelin receptors and has been considered as a therapeutic option in COPD patients with PH [103]. However, in a double blind placebo-controlled study patients with severe COPD treated with Bosentan for 12 weeks have failed to show significant improvement in exercise capacity. Conversely, they were found to develop worsening hypoxemia and reduced functional status [104]. This finding may have been a reflection of short treatment duration, with the effects of Bosentan underestimated. Held and Jany treated 4 COPD patients with long-term Bosentan and found maximum gains in 6 min walk distance after 9, 13, and 18 months of treatment, with no side effects or change in oxygenation [105].

Bosentan has also been used in the treatment of patients with PH and IPF with variable results. King et al. [106] evaluated the effect of Bosentan on 6MWT and quality of life (QOL) in patients with IPF. While no significant improvement in 6MWT compared to placebo was observed, assessments of dyspnoea and QOL favoured treatment with Bosentan. Although Bosentan was well tolerated, it could not delay progression of IPF or death [107].

Ambrisentan, a selective ETa receptor antagonist, has been used successfully in one case report of a patient with combined COPD and IPF and resulted in sustained improvement after 9 months [108]. However, these findings were not replicated in the ARIES-3 trial, which showed no effect in 6MWT distance either in COPD or ILD patients with PH after 24 weeks of treatment [109].

### 7.6. Prostacyclin

Prostacyclin is a vasodilator secreted from endothelial cells. Different forms of prostacyclins have been used in the treatment of patients with PH with varying success. Early studies of the effects of intravenous prostaglandin E1 (PGE1) observed decreases in pulmonary vascular resistance with increased cardiac output but were associated with worsened V/Q mismatch and increased hypoxemia [110]. In a recent case study Shimizu et al. [111] reported significant haemodynamic improvement in a patient with disproportionate PH using long-term intravenous infusion of epoprostenol for over 3 years. A more recent study in patients with advanced IPF and PH showed that PH-targeted therapy with trepostinil may improve right heart haemodynamics and echocardiographic function without affecting systemic oxygen saturation [112].

Inhaled prostaglandins, whose localized delivery targets only well-ventilated areas of lung, reduce V/Q mismatch while minimizing systemic distribution and associated side effects. Results of treatment with inhaled Iloprost, a prostacyclin analogue, have been controversial. In a randomized controlled crossover study of 16 patients with COPD-related PH, Iloprost failed to improve 6MWT as compared to placebo and was associated with impaired oxygenation at rest [91]. However, in a further study involving 10 COPD patients with PH, low dose inhaled Iloprost was seen to improve gas exchange and exercise tolerance 30 minutes after administration [92].

Park et al. performed a systematic review and meta-analysis of the effect of PH-specific therapy on exercise capacity in stable COPD patients [113]. They included 4 RCTs involving 109 subjects treated for more than 6 weeks with Bosentan, sildenafil, and Beraprost. The pooled analysis showed significant improvement in exercise capacity in COPD patients with overt PH at rest (mean difference 111.6; 95% CI: 63.3–159.9). However, a similar effect was not observed in patients with no proven PH on RHC. There was no significant worsening of hypoxemia with treatment in this meta-analysis [113].

In general, the effects of PH-specific vasodilators on WHO class 3 PH patients with CLD are still controversial with no significant demonstratable improvement in outcomes such as mortality or time to clinical worsening. While small studies and case cohorts seem to suggest some favourable impact in patients with disproportionately severe PH, additional larger, well designed, long-term clinical trials are needed to clarify the efficacy and safety of such PH therapies in patients with CLD.

### 7.7. Calcium Channel Blockers

Calcium channel blockers, as nonselective vasodilators, have been used for the treatment of PH. Their use has been debatable due to reports of worsening V/Q mismatch [114, 115], lack of long-term effectiveness through the development of tolerance [116, 117], and the high incidence of side effects including ankle oedema, headache, and facial flushing [117]. In our earlier study felodipine, a nonselective dihydropyridine calcium channel blocker, significantly improved pulmonary haemodynamics in patients with COPD and PH [118]. Pulmonary vasodilatation in these patients was sustained for 3 months of treatment without development of tolerance or deterioration in gas exchange, although a high incidence of vasodilator side effects was observed. Our subsequent study revealed amlodipine to be as effective as felodipine in improving pulmonary haemodynamics in patients with COPD, with fewer side effects [119]. One small RCT in patients with COPD and PH reported significant improvement in dyspnoea score as well as preserved cardiac output after 1 year of nifedipine treatment; however, no significant survival benefits were reported [120]. Such evidence nevertheless supports the hypothesis that pulmonary vasodilatation in patients with severe COPD and PH may improve functional performance, dyspnoea, and QOL, particularly if systemic vasodilatation side effects can be avoided.

### 7.8. Statins

Apart from cholesterol lowering and immune-modulating effects, statins have been found to reduce the level of ET1 [121] and improve PH in COPD patients [122]. In a double-blind placebo-controlled trial of 53 COPD patients with PH who were treated with Pravastatin for 6 months there was significant improvement in exercise capacity, dyspnoea scores, and pulmonary pressures through ET1 synthesis inhibition [122]. These results were not replicated in a similar study, which found no significant changes in systolic PAP, RV size, CO, or exercise capacity following 6 months of statin therapy [123].

### 7.9. Riociguat

Riociguat is a stimulator of soluble guanylate cyclase (sGC), a molecule that binds to NO and stimulates cGMP. It has a favourable safety profile and improves exercise capacity, symptoms, and pulmonary haemodynamics in PAH and chronic thromboembolic PH [124]. In an open-label uncontrolled pilot trial, 22 patients with ILD induced PH received oral Riociguat for 12 weeks. Riociguat was well tolerated by most patients and improved cardiac output and PVR, but not mPAP [125].

### 7.10. Lung Volume Reduction Surgery (LVRS) and Transplantation

Despite early results of LVRS, which showed improvement in LV end-diastolic dimension and filling [126], subsequent studies have failed to show any haemodynamic changes postoperatively as compared to subjects who were treated medically [127]. Lung transplantation is a last-resort procedure for suitable patients with advancing CLD and RHF. The appropriate timing of transplantation has been debated and is discussed in a recent review article [128].

## 8. Conclusion

PH is relatively common in CLD and often progresses to RHF. Mechanisms may differ in various disorders and may include pulmonary vasoconstriction, pulmonary vascular remodelling, small vessel destruction, and fibrosis. Currently, there are no specific therapies approved for management of PH or RHF in CLD. Therefore, treatment is aimed at treating the underlying lung disorder and/or hypoxia, as well as managing fluid balance. More translational research and prospective studies are needed in this area.
